# DNA Methylation as a Noninvasive Epigenetic Biomarker for the Detection of Cancer

**DOI:** 10.1155/2017/3726595

**Published:** 2017-09-05

**Authors:** Catherine Leygo, Marissa Williams, Hong Chuan Jin, Michael W. Y. Chan, Wai Kit Chu, Michael Grusch, Yuen Yee Cheng

**Affiliations:** ^1^Asbestos Disease Research Institute, Sydney Medical School, University of Sydney, Rhodes, NSW, Australia; ^2^Biomedical Research Center, Sir Runrun Shaw Hospital, Zhejiang University, Hangzhou, China; ^3^Institute of Molecular Biology, National Chung Cheng University, Min-Hsiung, Chia-Yi, Taiwan; ^4^Department of Ophthalmology and Visual Sciences, The Chinese University of Hong Kong, Shatin, Hong Kong; ^5^Institute of Cancer Research, Department of Medicine I, Medical University of Vienna, Vienna, Austria

## Abstract

In light of the high incidence and mortality rates of cancer, early and accurate diagnosis is an important priority for assigning optimal treatment for each individual with suspected illness. Biomarkers are crucial in the screening of patients with a high risk of developing cancer, diagnosing patients with suspicious tumours at the earliest possible stage, establishing an accurate prognosis, and predicting and monitoring the response to specific therapies. Epigenetic alterations are innovative biomarkers for cancer, due to their stability, frequency, and noninvasive accessibility in bodily fluids. Epigenetic modifications are also reversible and potentially useful as therapeutic targets. Despite this, there is still a lack of accurate biomarkers for the conclusive diagnosis of most cancer types; thus, there is a strong need for continued investigation to expand this area of research. In this review, we summarise current knowledge on methylated DNA and its implications in cancer to explore its potential as an epigenetic biomarker to be translated for clinical application. We propose that the identification of biomarkers with higher accuracy and more effective detection methods will enable improved clinical management of patients and the intervention at early-stage disease.

## 1. Types of Noninvasive Epigenetic Biomarkers

### 1.1. The Need for Biomarkers in the Diagnosis of Cancers

Novel diagnostic and prognostic biomarkers are urgently needed to aid in the prevention and management of cancers worldwide. The detection of aberrantly expressed biological molecules manifested during carcinogenesis can serve as a guideline for clinicians to make informed judgments based on predicted variables such as the likelihood of metastasis, tumour recurrence, and expected length of patient survival [1]. Currently, there is a shortage of noninvasive biomarkers with sufficient accuracy to identify patients in need of treatment, especially during the early stages of cancer where intervention has the highest potential to reduce mortalities [2]. Unfortunately, cancer diagnosis is complex and is often confounded by issues such as the long latency periods of some tumours and lack of clinical presentation at the early stages of disease [1, 3]. As a result of this, delayed intervention is a frequent occurrence, which facilitates the progression into more advanced stages of cancer. Therefore, the discovery and development of novel biomarkers are urgently needed for the screening of high-risk populations to enable prompt and successful treatment [1].

### 1.2. Limitations of Current Detection Methods for Cancer

Currently, there are numerous noninvasive techniques for the detection of specific cancer types including computed tomography (CT) in lung cancer, mammography in breast cancer, or positron emission tomography (PET) scan and digital rectal examination (DRE) for prostate cancer [4–6]. Many imaging techniques have a high sensitivity for the detection of abnormal neoplasms but lack the capacity to classify tumour subtypes or distinguish benign from malignant tumours [7]. In breast cancer screening, the use of mammography is limited to the detection of larger tumours, resulting in the neglect of smaller tumours [8], and diagnosis is dependent on the level of expertise possessed by the clinician [6]. In prostate cancer, as part of the physical examination of the prostate gland, the DRE is semi-invasive and can cause injury and bleeding to the patient [4]. The current gold standard for cancer diagnosis is histopathology; this method is dependent on invasive and often painful techniques. Techniques such as fine needle aspiration (FNA) and a core biopsy are required for the extraction of suspected tumour tissue and subsequent histological evaluation [9]. Histopathological assessment has its limitations, and the accuracy of results is dependent on the quality and yield of tissue obtained. For example, only 0.03% of tissue from the entire prostate is removed during core needle biopsy and may not accurately represent the actual tumour core when assessing for prostate cancer [10]. There is also an inherent risk of physical damage to functional organs during biopsy, for example, the possibility of pneumothorax during the investigation of respiratory neoplasms [11]. A further limitation of histological analysis is that tissue analysis is subject to the interpretation of the pathologist, which can be unreliable in the case of rare cancers or cancers with ambiguous histological features [12]. Therefore, it is imperative that for the purposes of histopathology, a representative section of tumour status is obtained; however, this cannot be easily guaranteed when retrieving tumour tissue during biopsy [10].

### 1.3. The Impact of DNA Methylation in Carcinogenesis

The most widely studied epigenetic alteration to date is the methylation (5-methylcytosines) of DNA at the CpG dinucleotides, which are highly concentrated in the CpG islands within the promoter region or near the first exon [13]. Varying degrees of methylation within a gene's CpG islands leads to various levels of gene silencing, and in cancer, promoter hypermethylation has been linked to the silencing of tumour suppressor genes and subsequent oncogenesis [14–16]. Screening for gene mutations is a common practice to test for an individual's predisposition to cancer but cannot reflect the current status or activity of disease [17]. Additionally, promoter methylation is often easier to evaluate due to its defined location within the promoter region of specific genes. Comparatively, locating gene mutations is more complex as they can occur at unsuspected sites within the gene that may be challenging to pinpoint. Some epigenetic markers have value in the early detection of cancers due to their involvement in the initiation of carcinogenic pathways [18, 19]. As a consequence, epigenetic biomarkers have a high potential and wide scope to be implemented as early diagnosis biomarkers.

### 1.4. Noninvasive Epigenetic Biomarkers for Cancer

Despite the benefits of current noninvasive detection methods for the screening of cancer, the accuracy of results is still limited. To achieve a conclusive and accurate diagnosis, the use of invasive techniques are necessary. Epigenetic biomarkers can be extracted using noninvasive techniques to determine prognosis and accurately predict the outcomes of disease [20]. Recent developments in epigenetics have explored the use of biological fluids including blood-based biomarkers, which are under investigation for their potential to limit the need for biopsy [21]. A good source of tumour-derived nucleic acids is peripheral blood, which can be retrieved noninvasively through venipuncture also referred to as liquid biopsy [22]. Circulating cell-free DNA can be isolated from plasma or serum for the evaluation of epigenetic changes. One of the major areas in epigenetic-based biomarker research is promoter methylation which can be detected in DNA extracted from bodily fluids such as serum, saliva, and urine.

### 1.5. Types of Noninvasive Biospecimens for the Detection of Methylated DNA

Individual cancers, depending on their anatomical location, have characteristic mechanisms for shedding tumour DNA into closely related bodily fluids. These biological fluids can be exploited as a source for biomarker investigation. For example, urine and urinary sediment can harbor carcinoma cells that are not accessible through biopsy from bladder cancer [23–25] or prostate cancer [10]. Therefore, urine represents an attainable source of tumour-derived DNA that is easily excreted and collected in a noninvasive manner [26–28]. Sputum can possess malignant cells from lung cancer and has been determined to provide a more accurate methylation status compared to blood-based samples [29–32], as reported when observing a salivary rinse for oral cancer [33]. This is a result of the copious amounts of DNA shed from the tumour cells from the thoracic and oral regions into sputum and saliva. Stool can also be used to detect tumour-derived methylated DNA biomarkers for colorectal cancer [34–36] and pancreatic cancer [37]. A large proportion of the sample types evaluated to detect circulating cell-free biomarkers are blood based [38, 39] in the detection of many cancers [40, 41] as it contains a high volume of genetic material. Comparatively, plasma provides a more accurate representation of circulating cell-free DNA for the detection of cancers to serum, which can contain DNA contaminations as a result of coagulation [42].

## 2. Methods for the Detection of DNA Methylation in Tissue and Biological Fluid

There are numerous methods that can be applied for detection of epigenetic biomarkers, which encompass whole genome screening, pyrosequencing, quantitative methylation-specific PCR (qMSP), MethyLight assay, and one-step methylation-specific polymerase chain reaction (OS-MSP) assay [43–47]. The variety of techniques that have been developed to detect DNA methylation each has its own advantages and limitations [48]. Genome-wide methylation sequencing or microarray-based profiling is often used to identify candidate biomarkers, whereas the performance of a specific marker or a limited panel of markers in larger cohorts is typically assessed using locus-specific assays such as quantitative methylation-specific PCR (qMSP), one-step methylation-specific PCR, MethyLight assay, and pyrosequencing, which can detect methylation of known loci with high sensitivity and specificity [43–45, 49, 50].

## 3. Noninvasive Epigenetic Markers in Cancers (Table 1)

### 3.1. Prostate Cancer

Prostate cancer is amongst the most frequently diagnosed cancers in the world, affecting 31.1 per 100,000 men [1] and accounting for 1 death every 4 minutes [51]. Determining the prognosis of prostate cancer is difficult due to the lack of accuracy in the biomarkers currently available [21]. The developments of noninvasive detection biomarkers for prostate cancer will largely facilitate the management of this cancer. Serum prostate-specific antigen (PSA), the conventional marker used to diagnose prostate cancer, is upregulated in individuals with the disease; however, it has poor sensitivity and specificity as an individual marker [21]. A number of genes with tumour suppressor functions have been assessed for epigenetic changes in prostate cancer to provide an alternative biomarker to PSA. Brait et al. recently used qMSP to test 10 genes previously associated with methylation in prostate cancer tissue and developed a panel of 3 methylated genes to assist in the detection of prostate cancer [21]. In this study, serum samples from 84 prostate cancer patients, 30 cancer-free controls, and 7 patients with the precancerous prostate abnormality high-grade prostatic intraepithelial neoplasia (HGPIN) were evaluated for promoter methylation [21]. Of the 10 genes tested, the methylation status of *SSBP2*, *MCAM*, *ERα*, *ERβ*, *CCND2*, *MGMT*, *GSTP1*, and *p16* genes were best matched when comparing the results from serum to prostate cancer tissue [21]. *MCAM* methylation was most accurate (with AUC of 0.66 obtained from the ROC), being detected in 85% of the early-stage (T1c) cancers (*n* = 60). When combining *MCAM* methylation with PSA threshold of >4 ng/ml [21], there was 91% detection of early-stage cancers. Finally, detection of at least one methylated gene in a panel of 3 genes, *MCAM*, *ERα*, and *ER*, improved specificity to 70% in comparison to serum PSA, which was only 30% [21]. Deng et al. used qMSP and found that hypermethylation in serum promoter *protocadherin 10* (*PCDH10*) was associated with worse prognosis and shorter survival from patients undergoing preporostate transurethral resection [22]. They included 171 prostate cancer patient samples and 65 controls with benign prostatic hyperplasia and found 51.5% of hypermethylation in cancer correlated to preoperative high PSA level (*p* = 0.001) [22], worse prognosis, and lymph node metastasis [22]. Similarly, Wang et al. showed that methylated serum *CDH13* was detected in 44.9% of 98 prostate cancer samples (with shorter survival) and undetectable in 47 control serum [52]. Increased *CDH13* promoter methylation was also associated with higher PSA levels [52]. Both studies indicated no methylation of the *PCDH10* and *CDH13* in benign prostate controls.

The most frequently studied epigenetic marker in prostate cancer is *Glutathione S-transferase 1* (*GSTP1*) [53]. It is methylated in prostate cancer tissue and most prostate cancer cell lines [54]. Recently, *GSTP1* promoter methylation has been evaluated for its potential as a noninvasive epigenetic marker in peripheral blood [41] and urine [10, 53, 55]. *GSTP1* is commonly methylated in prostate cancer tissue, blood, and urine, which has been confirmed in multiple studies [10, 53, 55]. *GSTP1* methylation in plasma was detected using qMSP in a phase I exploratory cohort of 75 men and further validated in an independent cohort of 51 men [41]. *GSTP1* hypermethylation was associated with poor prognosis and poor overall survival and was a good predictor for worse prognosis after treatment with chemotherapy [41].

Daniunaite et al. defined a panel of methylated promoter genes, which included *GSTP1*, *RASSF1*, and *RARB* for the detection of prostate cancer in urine [55]. One or more of the 3 genes were detected using qMSP in 82% of 37 catheter urine samples from patients with prostate cancer [55]. Jatkoe et al. found that hypermethylation of GSTP1 and APC in urine was a highly sensitive biomarker for early diagnosis using a cohort of 665 prostate cancer patients. The methylated gene combination was also more representative of disease status than Gleason score used to analyse biopsy tissue [10]. A study by Woodson et al. evaluated *GSTP1* methylation as an independent biomarker in urine with a 75% sensitivity and 98% specificity rate in urine compared to the 88% specificity and 91% sensitivity of prostate cancer tissue specimens [53]. The detection of *GSTP1* promoter methylation in urine was significantly higher in stage III at 100% when compared to 20% in stage II samples (*p* = 0.05) [53].

### 3.2. Bladder Cancer

Bladder cancers are a rapidly progressing illness with high prevalence and varied symptoms from patient to patient [56]. Patients with previous disease require persistent screening posttreatment, based on a high risk of tumour recurrence [57]. The current gold standard for diagnosis is cystoscopy which is invasive, and its high cost and complexity render this method inappropriate for repetitive screening [23, 24]. Urine in bladder cancer contains cancer cells exfoliated from the epithelial lining of the bladder containing tumour-derived DNA. Studies have successfully identified urine as a source for detection of epigenetic modifications [23, 24]. Several studies have evaluated methylation of multiple tumour suppressor genes to determine their relationship with bladder cancer tissue and subsequent methylation status in urine, and some of these genes include *APC*, *ARF*, *BAX*, *BCL2*, *CDH1*, *CDKN2A*, *DAPK*, *EDNRB*, *EOMES*, *FADD*, *GDF15*, *GSTP1*, *LITAF*, *MGMT*, *NID2*, *PCDH17*, *POU4F2*, *RARβ2*, *RASSF1A*, *TCF21*, *TERT*, *TIMP3*, *TMS-1*, *TNFRSF21*, *TNFRSF25*, and *ZNF154* [24, 45, 58].

Wang et al. studied the combination of hypermethylated *POU4F2* and *PCDH17* and found 90% sensitivity and 93.6% specificity in a cohort of 312 individuals using qMSP [24] when compared to healthy controls and other pathological bladder conditions including infected urinary calculi, kidney cancer, and prostate cancer [24]. Similarly, a study by Hoque et al. confirmed an increase in *CDKN2A*, *ARF*, *MGMT*, and *GSTP1* methylation status to correlate with tumour progression whilst in healthy control samples, methylation was undetectable when using the qMSP technique [58]. Friedrich et al. studied *DAPK*, *BCL2*, and *TERT* and found that they were methylated in bladder cancer when comparing to 20 healthy controls [45]. Hypermethylation of *TERT* and *BCL2* was associated with tumour grading, and *BCL2* was associated with tumour stage [45]. Utilising a microdroplet-based PCR, a high-throughput next-generation sequencing approach targeting DNA methylation, Feber et al. developed a 150 CpG loci test called UroMark [23]. This genome-wide methylation profile detected bladder cancer with high accuracy by receiver operator characteristic (ROC) and achieved 0.97 of area under the curve (AUC) from overall 167 noncancer controls and 107 bladder cancer samples [23].

### 3.3. Colorectal Cancer

Colorectal cancer has the 3rd highest global prevalence of cancers with 1.9 million men and 1.6 million women affected, and its incidence is more common in developed countries, recorded by WHO in 2014 [1]. Due to the high recurrence rates of colorectal cancer, there is an urgency to develop noninvasive biomarkers appropriate for frequent screening [59]. The current major form of diagnosis for colorectal cancer is through colonoscopy which is an invasive and time-consuming method [60]. Aberrant methylation of genes can be used in such a way that they reflect cancer stage [17] and subsequently exploited to aid clinicians as diagnostic or prognostic biomarkers.

Various genes related to malignant development have been investigated in the tissue and bodily fluids of colorectal cancer patients including *Fibrillin-2* (*FBN2*), *MAL*, septin 9 (*SEPT9)*, tachykinin-1 (*TAC1*), nel-like type 1 (*NELL1*), cellular retinoic acid binding protein 1 (*CRABP1*), somatostatin (*SST*), eyes absent homolog 4 (*EYA4*), and *Vimentin* (*VIM*) [59, 61, 62]. The current biomarkers used in the clinic for detection of colorectal cancer are carcinoembryonic antigen (CEA) and CA19-9 which have limited accuracy for independent diagnosis [63]. The following studies have compared the validity of these genes as potential biomarkers in colorectal cancer.

Serum hypermethylation of the *SST* and *MAL* genes were confirmed by Liu et al. in a study including 165 preoperative stages II and III colorectal cancer patients [59]. Hypermethylation was correlated to high recurrence of tumours in a 56-month median follow-up study of serum *SST* and *MAL* [59]. In a similar study, Tham et al. identified increased methylation of *TAC1*, *SEPT9*, and *NELL1* genes in serum to be correlated to poor prognosis in colorectal cancer in a cohort of 150 patients [63]. This study concluded that hypermethylation of *TAC1* and *SEPT9* promoter regions was detectable earlier in patients postresection compared to CEA and was a better predictor of tumour recurrence [63]. Additionally, it was found that the risk of cancer-specific mortality increased with hypermethylation of the *NELL1* promoter region [63]. Herbst et al. investigated the preoperative serum of 106 colorectal cancer patients followed by curative resection and revisited in a 5-year follow-up study [20]. This research found *HLTF* methylation to be significantly higher in patients that experienced tumour recurrence (*p* = 0.014) [20]. Therefore, the detection of methylation in the genes *SST*, *TAC1*, *SEPT9*, and *HLTF* may enable early detection and assist in more effective intervention during postresection tumour recurrence compared to conventional markers. Of these genes, *SEPT9* has recently obtained FDA approval for use as a noninvasive methylation marker in the clinic [64] and is distributed as the commercial test (Epi proColon test).

Silencing of *VIM* by promoter methylation has been linked to poor prognosis in colorectal cancer using qMSP to detect serum methylation level in several studies [61, 65]. *VIM* promoter methylation was tested in conjunction with CEA and carbohydrate antigen (CA 19-9) [65], which are elevated in the more advanced stages of colorectal cancer [66]. In the early stages (0–II) of colorectal cancer, detection of *VIM* methylation had a higher sensitivity than CEA and CA 19-9 [65]. When we combined results from 3 serum markers, the sensitivity was significantly increased to 85.7% for detection of patients with stage IV tumours compared to earlier stage cancers [65]. In a study by Shirahata et al., hypermethylation of *VIM* was detected in only 9% of the 44 serum samples from colorectal cancer patients [61]. However, when assessed in conjunction with clinicopathological features, *VIM* methylation was significantly increased in patients with advanced disease who had developed distant metastasis (*p* = 0.0063), secondary tumours in the liver (*p* = 0.026), and peritoneal dissemination (*p* = 0.0029) [61]. In a further study, the *Fibrillin-2* (*FBN2*) gene was determined to be hypermethylated in colorectal cancer tissue [62]. This was later confirmed in the serum of colorectal cancer patients with hepatic metastasis (*p* < 0.0001) and found at higher frequency in male patients [62]. The fecal occult blood test (FOBT) is used in the conventional testing for the detection of colorectal cancer in which stool is analyzed for the presence of blood from bowel lesions [34]. Chen et al. tested the results from FOBT in combination with *SEPT-9* hypermethylation in plasma [34]. A more accurate diagnosis for colorectal cancer was obtained yielding an AUC of 0.766 when compared to each technique performed individually [34].

Epigenetic changes associated with malignant bowel tissue have been detected in the stool of patients with colorectal cancer, rendering it a source for biomarker detection [35]. Li et al. determined that the presence of hypermethylation in at least one of the genes *SNCA* and *FBN1* was found to have a sensitivity and specificity of 84.3% and 93.3%, respectively [35]. This study examined 89 stool samples from colorectal cancer patients and 30 healthy controls using qMSP [35]. Glockner et al. discovered that tissue factor pathway inhibitor 2 (*TFPI2*) was methylated in 99% of 115 stool samples from colorectal cancer patients [67]. From the early to advance stages, sensitivity increased from 76% to 79% and specificity from 89% to 93%, suggesting the use of methylated *TFPI2* with a good accuracy for screening from the early stages of carcinogenesis [67].

### 3.4. Lung Cancer

Lung cancer is a commonly diagnosed and aggressive respiratory tumour with poor survival [1]. There are numerous subtypes to the disease with risk factors including age, tobacco smoking, and family history [1]. Initial screening for respiratory lesions is performed by computed tomography (CT), which is highly sensitive for the detection of lung cancer, but is subject to a high rate of false-positive results from the detection of benign tumours [7, 68]. There are serveral circulating biomarkers under investigation for screening of lung cancers; however, none exist with the accuracy to eliminate biopsy and histopathology for the final diagnosis [69]. Transmembrane protein with a single EGF-like and two follistatin domains (*TMEFF2*) is inactivated through promoter methylation in numerous cancers including non-small-cell lung cancer (NSCLC) [70]. Hypermethylation of *TMEFF2* is more common in non-*EGFR* mutation female patients (*p* = 0.06) and subjects who have never smoked (*p* = 0.07) [70]. In this study, serum samples from 316 NSCLC patients and 50 healthy age-matched controls were used and *TMEFF2* hypermethylation was detectable in 9.2% of NSCLC cases and no methylation in healthy controls [70]. Hypermethylation of *RASSF1A* was detected in 33.8% of NSCLC and not in healthy controls of benign pulmonary disease in the cohort of 80 patients [14].

Sputum as a biospecimen is more representative of NSCLC compared to blood due to the direct shedding of tumour DNA from the lung [14, 71]. Belinsky et al. used sputum and serum to identify methylation targets for NSCLC and found that *p16*, *DAPK*, *PAX5b*, and *GATA5* were potential biomarkers for NSCLC [71]. They included 72 stage III NSCLC patients to compare tumour tissue, sputum, and serum [71]. Methylation status was higher in sputum and similar to tumour tissue but low in serum [71]. Su et al. analysed microRNA and DNA methylation in the cohort of 117 stage one NSCLC patients and 174 healthy smokers. Results indicated methylation of *RASSF1A*, *PRDM1*, and *3OST2* were useful for early detection at stage 1. They also found that miR-21, miR-31, and miR-210 were sensitive for early detection in between 62% and 77% of cases [29]. Even though these genes could facilitate cancer detection at earlier stages, further validation of the results is required to ensure clinical relevance. Palmisano et al. reported that DNA methylation of *MGMT* was detectable in squamous cell lung carcinoma 3 years before clinical diagnosis [72]. Miglio et al. detected *MGMT* promoter methylation in 1 sample of sputum and 6 out of 8 bronchial washings from patients with small-cell lung cancer (SCLC) [73]. Agustí et al. reported that the induced sputum from peripheral lung cancer patients provided specimens of higher integrity and better diagnostic value [74].

### 3.5. Breast Cancer

Breast cancer is the most frequently diagnosed cancer in women, with the highest incidence (43.3 per 100,000) in women [1]. It affects individuals at a younger age compared to other majorly diagnosed cancers [1]. Conclusive detection of breast cancer is carried out using mammography, which has a sensitivity and specificity greater than 70% and 85% with larger tumours [8]. However, if the tumour is less than 1 cm, the sensitivity declined and epigenetic biomarkers could facilitate diagnosis in this subgroup [8].

Visvanathan et al. developed an epigenetic gene panel of 6 genes: *AKR1B1*, *HOXB4*, *RASGRF2*, *RASSF1*, *HIST1H3C*, and *TM6SF1* to predict survival in the early stages of metastatic breast cancer [75]. The serum from 141 women with metastatic breast cancer showed higher levels of methylation in patients with longer median progression free and overall survival [75]. Yamamoto et al. established a more efficient method to detect DNA methylation in serum using the one-step methylation-specific polymerase chain reaction (OS-MSP). They found promoter methylation of *GSTP1*, *RASSF1A*, and *RARb2* using 101 patients with primary breast cancer, 58 with metastatic breast cancer, and 87 healthy controls. They also determined higher sensitvity of these markers in early-stage primary tumour when compared with the conventional markers CEA and/or CA15-3. The combination of conventional markers with the panel of three epigenetic markers has an improved sensitivity to detect metastatic breast cancer [44]. Shan et al. developed a 6-gene panel using MethyLight to test for methylation in serum [8] and found that *SFN*, *hMLH1*, *HOXD13*, *PCDHGB7*, *RASSF1*, and *P16* were methylated in breast cancer patient serum [8]. Promoter methylation in this panel was correlated to patients with a family history of tumours and inversely correlated with proliferative index (ki-67). This study included serum from 268 patients with breast cancer, 236 patients with benign breast abnormalities, and 245 healthy volunteers [8]. Liu et al. reconfirmed *BRCA1* was not significantly methylated in breast cancer serum samples; however, hypermethylation of the *FHIT* was significantly higher in individuals with breast ductal carcinoma compared to healthy controls and those with benign breast tumours [76]. This study assessed gene methylation using the bisulfite sequencing method and high-resolution melting curve analysis in the serum of 36 patients with breast ductal carcinoma, 30 with benign breast fibroadenoma and 30 healthy volunteers [76]. Hagrass et al. studied a cohort of Egyptian women and discovered that promoter hypermethylation of the *estrogen receptor alpha* (*ERα*) was most frequently detected in individuals with breast cancer when tested in the serum of 120 patients with breast cancer compared to 100 benign breast lesions [77].

### 3.6. Ovarian Cancer

Ovarian cancer is a rare cancer affecting women and the most lethal gynecological malignancy [78, 79]. *RASSF1A* promoter methylation has been identified by numerous studies in the early stages of carcinogenesis in ovarian cancer tissue [80]. Giannopoulou et al. evaluated hypermethylation of *RASSF1A* in plasma and tissue samples collected from 53 patients with high-grade serous ovarian cancer, using real-time MSP with 62.3% of plasma samples showing a correspondence in *RASSF1A* methylation to matched ovarian cancer tissue [81]. Similarly, Wu et al. evaluated promoter methylation of *RASSF2A* and detected hypermethylation in 36.2% of plasma samples from ovarian cancer patients and hypermethylation in 51.1% in paired tissue samples, which was absent in plasma samples of 14 patients with benign disease and 10 normal controls [82]. Flanagan et al. identified that DNA methylation is promoted following platinum-based chemotherapy in the blood of 247 ovarian cancer patients and is associated with survival using methylation arrays and bisulfite pyrosequencing [83]. Current research has focused on epigenetic changes in malignant tissue, and there is a need for further investigation into noninvasive biomarkers for ovarian cancer.

## 4. Conclusion

Epigenetic biomarkers are a promising area of research with DNA methylation having the potential to provide a wealth of information regarding physiological and pathological status. Different stages and types of cancer produce a unique epigenetic signature. Methylation signatures can be implemented as specific and accurate biomarkers to establish tumour type and assist with prognosis and cancer management. Importantly, epigenetic markers can assist in the detection of cancers from the early stages, making them a favorable addition to the current set of detection methods used in the clinic. The studies reviewed here exemplify the recent research into DNA methylation including combinations of epigenetic markers which can produce an improved diagnostic power when compared to evaluating biomarkers individually. To facilitate the widespread use of epigenetic biomarkers in the clinic, the biomarkers in question and detection methods require standardisation for each cancer type. Currently, to the best of our knowledge, only *SEPT9* has received approval from the Food and Drug Administration (FDA) for use as a blood-based methylated biomarker for the diagnosis of colon cancer. Although other methylation-based biomarkers under investigation have been shown to have clinical relevance, further research is still necessary. These genes include *BRCA1* in breast cancer, *MGMT* in gliobastoma multiform (GBM), and *MLH1* in colon cancer. These biomarkers can enable the differentiation between tumour types in the clinic however still require invasive collection methods, and therefore, future developments of noninvasive methylation detection markers are needed. In conclusion, epigenetic alterations hold a great potential to become routine clinical cancer biomarkers due to their accuracy, specificity, and ease of collection, which justifies further research to implement standard panels of noninvasive epigenetic biomarkers to diagnose different malignancies.

## Figures and Tables

**Table 1:** Noninvasive epigenetic DNA methylation biomarkers used in cancers.

Methylated gene	Marker (Dx/Px)	Cancer	DNA source	Cohort	Method	Accuracy (statistic of individual gene)	Ctrl†	Accuracy of panel including methylated gene		Ref
								Genes (*n*)	Statistic	
*ARF*	Dx	Bladder	Urine sediment	15 BlCa, 25 NC	qPCR	†27%, (4/15)	100% -ve	4	∆82%/96%	[58]
*APC*	Px	Prostate	Urine	665 men with elevated PSA	qPCR	—	—	2	—	[10]
*BCL*	Dx	Bladder	Urine sediment	37 BlCa, 20 NC	MethyLight	†65% (24/37)	100% -ve	3	†78% (29/37)	[45]
*CDH13*	Px	Prostate	Serum	98 PCa, 47 NC	qMSP	†45%, 44/98	100% -ve	—	—	[52]
*CDKN2A*	Dx	Bladder	Urine sediment	15 BlCa, 25 NC	qPCR	†47%, 7/15	100% -ve	4	∆82%/96%	[58]
*DAPK*	Dx	Bladder	Urine sediment	37 BlCa, 20 NC	MethyLight	†22% (8/37)	100% -ve	3	†78% (29/37)	[45]
ER*α*	Dx	Prostate/breast (primary)	Serum	16 PCa/120 BCa, 100 BN	qMSP	†13% (2/16), †72%	—	3	∆75%/70%	[21, 77]
*ERβ*	Dx	Prostate	Serum	16 PCa	qMSP	†13% (2/16)	—	3	∆75%/70%	[21]
*FBN1*	Dx	Colorectal	Stool	89 CRC, 30 NC	qMSP	†70.8% (63/89	93% -ve	2	∆84.3%/93.3%	[35]
*FBN2*	Dx	Colorectal (primary)	Serum	78 CRC	qMSP	†8% (4/49)		—	—	[62]
*GSTP1*	Dx, Px	Bladder/prostate/castrate-resistant prostate/breast	Urine sediment/urine/plasma/serum	15 BlCa, 25 NC/665 men w/ elevated PSA/34 PCa/24 PCa, 8HGPIN, BN/75 CR-PCa/120 BCa/101 BCa (primary), 58 BCa (secondary), 87 healthy	qPCR, OS-MSP	†47% 7/15/†4% 4/101/†3% 1/34/†67% 50/75	100% -ve	4, 2, 3, 3, 3	∆82%,96%/−/†82% (28/34)/∆75%/98%/†6% 7/120/†22% 22/101	[10, 41, 43, 44, 53, 55, 58]
*FHIT*	Dx	Ductal breast cancer	Serum	36 BCD, 30NC, 30 BN	qMSP and high-resolution melting curve analysis	64%	35% NC, 64% BC	—	—	[76]
*hMLH1*	Dx	Breast	Serum	268 BCa, 245 NC, 236 BN	MethyLight	†28% 75/268	85.71% -ve NC, 77.97% -ve BN	6	AUC = 0.727 (BCa versus NC), AUC = 0.789 (BCa versus BN)	[8]
*HLTF*	Px	Colorectal	Serum	106 CRC	MethyLight	—	—	—	—	[20]
*HOXD13*	Dx	Breast	Serum	268 BCa, 245 NC, 236 BN	MethyLight	†37/268	97.55% -ve NC, 99.58% -ve NC	6	AUC = 0.727 (BCa versus NC), AUC = 0.789 (BCa versus BN)	[8]
*MCAM*	Dx (early)	Prostate	Serum	16 PCa	qMSP	†46% (12/16)	—	3	∆75%/70%	[21]
*MGMT*	Dx	Bladder/lung	Urine sediment/bronchial washing	15 BlCa, 25 NC/8 SCLC	qPCR/MSP	†27% 4/15, †75% 6/8	100% -ve	4	∆82%/96%	[52, 73]
*NID2*	Dx	Bladder (primary)	Urine	157 BlCa, 339 urological disorder	qMSP	∆94%, 91%	—	2	†94% (466/496)	[84]
*P16*	Dx	Breast	Serum	268 BCa, 245 NC, 236 BN	MethyLight	†22% 60/268	83.27% -ve NC, 84.32% -ve BN	6	AUC = 0.727 (BCa versus NC), AUC = 0.789 (BCa versus BN)	[8]
*PCDHGB7*	Dx	Breast	Serum	268 BCa, 245 NC, 236 BN	MethyLight	†148/268	*52.65% -ve NC, 54.66% -ve BN*	6	AUC = 0.727 (BCa versus NC), AUC = 0.789 (BCa versus BN)	[8]
*PCDH10*	Px	Prostate	Serum	171 primary PCa, 65 BN	qMSP	†51.5% (88/171)	100% -ve	—	—	[22]
*PCDH17*	Dx	Bladder	Urine sediment	58 BlCa, 20 IUC, 20 KCa, 20 PCa	qMSP	AUC = 0.813	75% -ve	2	∆90%/93.96%	[24]
*PHACTR3*	Dx	Colorectal	Stool	11 CRC, 20 NC	qMSP	†44% 4/9	100% -ve	—	—	[85]
*POU4F2*	Dx	Bladder	Urine sediment	58 BlCa, 20 IUC, 20 KCa, 20 PCa	qMSP	AUC = 0.921	94% -ve	2	∆90%/93.96%	[24]
*TERT*	Dx	Bladder	Urine sediment	37 BlCa, 20 NC	MethyLight	†51% (18/37)	100% -ve	3	†78% (29/37)	[45]
*TMEFF2*	Dx	NSCLC	Serum	316 NSCLC, 50 NC		†9.2% 29/36	100% -ve	—	—	[70]
*RARB*	Dx	Prostate	Urine sediment	34 PCa	qMSP	†44% (15 of 34)		3	†82%(28/34)	[55]
*RARβ2*	Dx	Breast	Serum	120 BCa//101 BCa (primary), 58 BCa (secondary), 87 healthy	OS-MSP	−, †12% 12/101	—	3, 3	†6% 7/120/†22% 22/101	[43, 44]
*RASSF1*	Dx	Prostate	Urine sediment	34 PCa	qMSP	†71% 24/34		3	†82% (28/34)	[55]
*RASSF1a*	Dx, Px	Breast/lung/ovarian	Serum	268 BCa, 245 NC, 236 BN/101 BCa (primary), 58 BCa (secondary), 87 healthy /120 BCa/90 LCa/59 HGSC	qMSP/OS-MSP/MSP, MS-HRMA	†17% 46/268, †7% 7/101 †33.8% 27/80, †25.4% 15/59	89.67% -ve NC, 91.95% -ve BN/100% -ve	6, 3	AUC = 0.727 (BCa versus NC), AUC = 0.789 (BCa versus BN)/†6% 7/120/†22% 22/101	[8, 43, 44]
*RASSF2A*	Dx	Ovarian	Plasma	47 EOC, 14 BN, 10 NC	MSP	†36.2% 17/47	100% -ve BN, 100% -ve NC	—	—	[82]
*SEPT9*	Px	Colorectal	Serum	150 CRC, 60 CRC	qMSP, Abbott MS-9 reagent kit and PCR	−, ∆47%/89%	—	—	—	[34, 63]
*SFN*	Dx	Breast	Serum	268 BCa, 245 NC, 236 BN	MethyLight	†74% 197/268	85.71%-ve NC, 77.97% -ve BN	6	AUC = 0.727 (BCa versus NC), AUC = 0.789 (BCa versus BN)	[8]
*SNCA*	Dx	Colorectal	Stool	89 CRC, 30 NC	qMSP	†70% 62/89	100% -ve	2	∆84.3%/93.3%	[35]
*SST*	Px	Colorectal	Serum	165 CRC	—	—	—	—	—	[59]
*TAC1*	Px	Colorectal	Serum	150 CRC	qMSP	—	—	—	—	[63]
*TWIST1*	Dx	Bladder (primary)	Urine	157 BlCa, 339 urological disorder	qMSP	∆88%, 94%	—	2	†94% (466/496)	[84]
*VIM*	Dx	Colorectal	Serum	44 CRC, 239 CRC	qMSP	†9% 4/44, †33% 78/239	—	—	—	[61, 65]

Dx: diagnosis; Px: prognosis; BN: benign controls; NC: noncancer controls; ∆: sensitivity/specificity; †: % of samples detected; AUC: area under the curve (0-1) for ROC (receiver operating characteristic); BlCa: bladder cancer; BCa: breast cancer; BCD: breast ductal carcinoma; CR-PCa: castrate resistant prostate cancer; CRC: colorectal cancer; HGSC: high-grade serous ovarian cancer; IUC: infected urinary calculi: KCa: kidney cancer: LCa: lung cancer, NSCLC: non-small-cell lung cancer; PCa: prostate cancer; SCLC: small cell lung cancer.
